# 17q12 Microdeletion Syndrome as a Rare Cause of Elevated Liver Enzymes: Case Report and Literature Review

**DOI:** 10.7759/cureus.32233

**Published:** 2022-12-05

**Authors:** Hasan M Isa, Layla I Salman, Zainab A Almaa, Mariam Y Busehail, Zahra A Alherz

**Affiliations:** 1 Pediatric Department, Salmaniya Medical Complex, Manama, BHR; 2 Pediatric Department, Arabian Gulf University, Manama, BHR

**Keywords:** bahrain, hnf1b gene, elevated liver enzymes, solitary kidney, 17q12 deletion syndrome

## Abstract

17q12 deletion syndrome is a rare autosomal dominant inherited condition. It results from de novo mutation and can occur without a family history. Hepatocyte nuclear factor-1 beta (HNF1B) and LIM homeobox 1 (LXH1) genes are the most common genes to be deleted in this syndrome. It has unique clinical characteristics involving multiple systems in the body. The most common presentations are usually renal involvement and maturity-onset diabetes of the young type 5 (MODY5). Genetic study is the golden tool to diagnose patients with this syndrome. Our case presents the unique clinical features of 17q12 deletion syndrome along with a literature review.

## Introduction

17q12 deletion syndrome is a rare autosomal dominant inherited condition affecting the long arm of chromosome 17 [1-4]. Most cases result from de novo deletion, as it can occur in patients with no family history of this disorder [2] and 17q12 deletion syndrome can include multiple gene mutations [2]. In this condition, 15 genes can be affected but the most common to be deleted are HNF1B and LHX1 [2]. HNF1B gene belongs to the homeodomain-containing family of transcription factors whose mutation contributes to renal, hepatic, pancreatic, and urogenital tract disorders as it is involved in the organogenesis of the previously mentioned organs [2,5,6]. HNF1B gene mutation is a cause of MODY5 and renal abnormalities [2,5,7,8] that should be considered during screening for the disease or after the diagnosis is made, especially if there is renal involvement [7,9]. The LHX1 gene is responsible for early brain development and cognitive functions [8]. Accordingly, signs and symptoms differ from one patient to another depending on the type of gene deleted [2]. This syndrome is characterized by a wide variety of clinical phenotypes including renal abnormalities, MODY5, genital tract anomalies, dysmorphic features, neurodevelopmental disorders, and neuropsychiatric disorders [1-3,10].

This case report discusses an 11-year-old female who was diagnosed with 17q12 deletion syndrome based on the detection of a solitary kidney and deranged liver function tests (LFTs) during her annual renal screening. This is the first case of this rare disease to be reported from Bahrain.

## Case presentation

The case discusses an 11-year-old Bahraini girl who was born at term via a normal vaginal delivery with a low birth weight of 2.3 kg. Her antenatal ultrasounds showed a small-sized left kidney. She was diagnosed to have left renal agenesis soon after birth. However, her renal functions were normal and she had no other medical issues apart from an allergy to shrimp. Developmental milestones were appropriate for her age. Yet, she had average intelligence, with an Arabic-word reading disability. Regarding the family history, her parents were non-consanguineous and healthy, but her grandmother had chronic kidney disease. The patient was followed up by a pediatric nephrologist on a regular basis.

**Figure 1:**
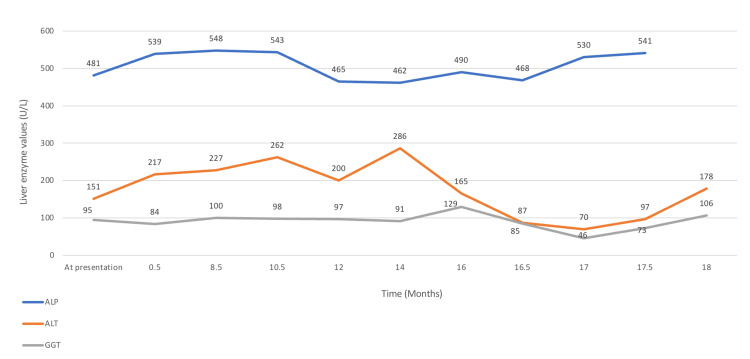
Liver enzymes values of an 11-year-old female patient with 17q12 deletion syndrome during her regular laboratory follow-up ALP, alkaline phosphatase (normal 150-480 U/L); ALT, alanine transaminase (normal< 33 U/L); GGT, gamma-glutamyl transferase (normal< 35 U/L).

At the age of nine years, she was referred to a pediatric gastroenterologist for frequent episodes of generalized body itching and elevation of her hepatic enzymes. Her physical examination was unremarkable without the presence of dysmorphic features or stigmatization of chronic liver disease. Liver enzyme values are shown in Figure 1. Ursodeoxycholic acid was prescribed for her which led to a significant reduction of both alanine transaminase (ALT) and gamma-glutamyl transferase (GGT) levels. However, ursodeoxycholic acid was discontinued after one month of administration as it led to the worsening of her itching symptoms.

The patient was thoroughly investigated for causes of elevated liver enzymes. Her complete blood count, glucose, urea, creatinine, serum fasting cholesterol, triglycerides, ceruloplasmin, alpha-fetoprotein, and alpha-1-antitrypsin levels were all within normal ranges. Viral serological markers (hepatotropic viruses, Epstein-Barr virus, and cytomegalovirus), auto-anti liver/gastrointestinal antibodies, and the total immunoglobulin levels were also unremarkable. Urine and stool tests showed normal results.

**Figure 2:**
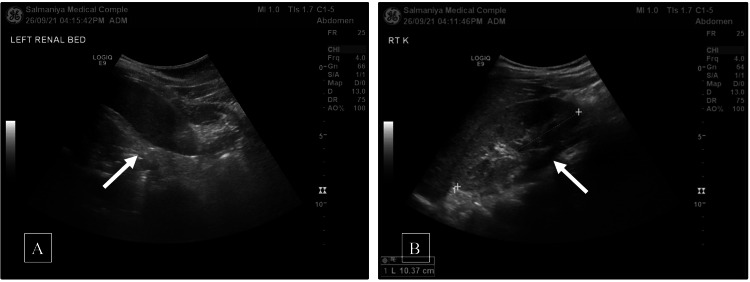
(A) Abdominal ultrasound of an 11-year-old female patient with 17q12 deletion syndrome showed empty left renal fossa (see arrow), and (B) normal right kidney in the same patient (see arrow).

Abdominal ultrasound showed left kidney agenesis (Figure 2). The right kidney was of normal size and echogenicity without any signs of hydronephrosis, presence of renal stones, or renal masses. The liver measured 14.4 cm showing homogeneous echotexture without the presence of definite focal lesions. The intra-and extra-hepatic biliary ducts were not dilated whereas the gallbladder, common bile duct, and spleen were normal.

At the age of 10 years, in view of the unclear cause of the elevated liver enzymes and the presence of renal agenesis, genetic testing was performed using next-generation sequencing (NGS)-based copy number variation (CNV) analysis. Chromosomal microarray analysis (CMA) was performed (CentoArrayCyto™- 750K, Centogene, Rostock, Germany) where 250 ng of genomic DNA were fragmented, amplified, and hybridized to the array according to the manufacturer's guidelines. The CytoScan 750K array (Affymetrix, Santa Clara, CA) contains 750,000 markers, including 200,000 single-nucleotide polymorphism (SNP) markers, across the whole genome covering 80% of the genes. By NGS-based CNV analysis, a 1534 kb one copy loss within chromosomal region 17q12 encompassing 33 genes including the HNF1B gene was detected and further confirmed by chromosomal microarray analysis. According to American College of Medical Genetics and Genomics (ACMG) guidelines, it has been classified as pathogenic. Deletion of similar size has been reported in cases with 17q12 recurrent deletion syndrome. Accordingly, this patient was fully investigated with regular outpatient follow-up and the parents were counseled.

## Discussion

Though 17q12 microdeletion syndrome is a rare condition, the exact prevalence of this syndrome is unknown due to undetected or unreported cases. The estimated prevalence is between 1 in 14,000 and 1 in 62,500 [2,8].

Despite being an autosomal dominant syndrome, upon reviewing the previously reported cases during the past 13 years, a slightly higher prevalence of 17q12 recurrent deletion syndrome among females was noted [3,5,8,6,11-15] (Table 1). Our patient was an 11-year-old female which is in line with the previously reported cases in terms of sex.

**Table 1:** Summary of previous studies of 17q12 deletion syndrome from other countries. Abbreviations: N, number; Y, year; Mg, magnesium; HNF1B, hepatocyte nuclear factor-1 beta; F, female; M, male; NR, not recorded; kb, kilobase; Mb, megabase; IQ, intelligence quotient; ADHD, attention deficit hyperactivity disorder; OCD, obsessive-compulsive disorder; MODY5, maturity-onset diabetes of the young type 5; del, deletion; dup, duplication; MRKHS, Mayar-Rokitansky-Kuester-Hauser syndrome; NODAT, new-onset diabetes mellitus after transplantation; ID, intellectual disability; MCDK, multicystic dysplastic kidney; DM, diabetes mellitus; INH, inherited; NA, not applicable; ASD, autism spectrum disorder; CKD, chronic kidney disease; ESRD, end-stage renal disease (a) Patient with additional gene mutations (AATF, CCL3L, C17orf78, DDX52, DHRSII, GGNBP2, LHX1, LOC284100, TBC1D3G, MRM1, MYO19, SYNRG, TADA2A, and ZNHIT3) (b, d) Patient with an additional gene mutation (LHX1) (c) Patient with additional gene mutations (LHX1 and COL3A1) (e) Both patients had additional gene mutations (PIGW, ACACA, HNF1B, ZNHIT3, GGNBP2, DHRS11, LHX1, AATF, TADA2A, DUSP14, DDX52, MYO19, MRM1, MRI2909, SNORA90, C17ORF78, SYNRG, MIR378) (f) Patient with additional gene mutations (CCL3L3, CCL3L1, CCL4L2, TBC1D3C, TBC1D3G, TBC1D3H, PIGW, ACACA, HNF1B, ZNHIT3, GGNBP2, DHRS11, LHX1, AATF, TADA2A, DUSP14, DDX52, MYO19, MRM1, MIR2909, SNORA90, C17ORF78, MIR378) (g) Patient with additional gene mutations (ZNHIT3, MYO19, PIGW, GGNBP2, DHRS11, MRM1, LHX1, AATF, MIR2909, ACACA, C17orf78, TADA2A, DUSP14, SYNRG, DDX52, LOC284100, TBC1D3F, TBC1D3, and LOC440434).

Country	Author/year	N	Age(Y)	Sex	Renal anomaly	Elevated liver enzymes	Low Mg	Others	HNF1B mutation
Bahrain	This case	1	11	F	Left renal agenesis	Yes	No	-	1534 kb deletion
Kingdom of Saudi Arabia	Roberts et al. [16] 2014	1	17	M	NR	No	No	Motor delay, social delay, low IQ, Autism, ADHD, OCD, behavioral problems, decrease sensitivity to pain, marfanoid habitus, skeletal defects, aortic root enlargement, hyperglycemia, small spleen, presence of accessory splenule	Deletion (de novo)^ a^
China	Cheng et al. [5] 2022	1	21	F	NR	No	Yes	MODY5, hypokalemia, non-atrophic gastritis with bile reflux.	1.85 Mb deletion
Japan	Nakamura et al. [17] 2021	1	Antenatal	M	Bilateral polycystic kidneys	No	No	-	1.35 Mb deletion (de novo)
Japan	Omura et al. [9] 2019	1	44	M	Left solitary kidney with multiple cortical cysts	Yes	Yes	Pancreatic hypoplasia, pancreatic exocrine dysfunction, MODY5, hypouricemia, hypercalciuria.	Deletion
Singapore	Hua Tan et al. [12] 2021	4	48	F	Right renal agenesis	yes	yes	MODY5	c.195_1868del p
			34	M	Multicystic kidneys	No	Yes	MODY5, hypokalemia.	c.195_1868del p
			29	M	Dysplastic right kidney & left renal cysts.	Yes	Yes	MODY5, congenital right lateral rectus palsy.	c.195_1868del p
			74	M	Left renal cysts	No	No	MODY	c.195_1868dup p
Taiwan	Chen et al. [11] 2013	1	Antenatal	F	Bilateral multicystic kidneys and hydronephrosis as well as dilated right renal calyces	No	No	-	1.75 Mb deletion ^b^
New Zealand	George et al. [3] 2011	1	7	F	NR	No	No	ADHD, disruptive behavior, learning difficulties, gross motor delay.	1.4 Mb deletion
Germany	Hinkes et al. [14] 2012	1	38	F	Right kidney aplasia and left kidney dysplasia.	No	No	MRKHS type II, NODAT, hyperelasticity, skeletal defects.	1.43 Mb deletion (de novo)^ c^
Italy	Palumbo et al. [1]2014	1	12	M	NR	No	No	Obesity, ADHD, OCD, ID, presence of dysmorphic features.	1.428 Mb deletion (de novo)
Italy	Bernardini et al. [15] 2009	2	20	F	Recurrent acute cystitis during infancy	No	No	MRKHS (primary amenorrhea, uterine agenesis) presence of mild dysmorphic features	1.5 Mb deletion (de novo)^ d^
			15	F	Bilateral multicystic kidneys	No	No	MRKHS (agenesis of the upper and middle third of the vagina, right unicornuate uterus)	1.5 Mb deletion (de novo)
Belgium	Raaijmakers et al. [6] 2014	20	Mean age 16	F>M	Bilateral renal cysts (n=14), renal dysplasia (n=10), hydronephrosis (n=2), MCDK (n=2), renal agenesis (n=1)	No	(n=6)	MODY(n=5), cholestasis (n=2), uterine anomalies (n=2), cerebellar abnormalities (n=1)	HNF1B deletion (n=10), HNF1B duplication (n=1), n=9 E1: c.226G>T (INH), E2:c.544C>T (INH), E6:c.1299delC (NA), E6: c.1253A>C (INH), E8:c.16640C>T (INH), E9:c.1654-2A>T (INH), E1: c.226G>T (INH), E3: c.684C>G (INH), E4: c.88C>T (de novo).
Germany	Roehlen et al. [8] 2018	2	16	F	Bilateral polycystic kidneys	Yes	No	DM, pancreatic atrophy, reduced intellectual performance	1.58 Mb deletion
			36	F	Multiple cysts at the lower pole of the left kidney	No	No	MODY5, pancreatic atrophy, with cystic formations in the residual of the pancreatic head, mild ID, presence of dysmorphic features	1.58 Mb deletion, 1.04 Mb duplication at 10q11.22
Germany	Vasileiou et al. [13] 2019	7	Antenatal	F	Bilateral renal cysts	No	No	Atrial septal defect	c.715-717del (de novo)
			NR	M	NR	No	No	Learning difficulties, oppositional behavior, ventricular septal defect	Deletion (de novo)
			12-15	M	Bilateral renal cysts	No	No	Mild ID, Asperger syndrome, hyperactivity, abnormal social behavior	Deletion
			NR	F	NR	Yes	No	Motor delay, learning and concentration difficulties, low-normal IQ, autistic-like behavior, enlarged brain ventricles, short stature, Brown’s syndrome	Deletion (de novo)
			14	F	Bilateral renal cysts, CKD stage II	No	No	Mild strabismus	Deletion (de novo)
			>26	F	Bilateral renal cysts, ESRD	Yes	No	Motor delay, single seizure attack in adulthood, biliary cirrhosis because of liver cysts	Deletion (de novo)
			4m	F	Right kidney agenesis, left kidney hypoplasia, ESRD	No	No	convergence strabismus, uterine aplasia, ovarian cysts, aortic insufficiency, joint hyperextension	Deletion (de novo)
Romania	Țuțulan-Cuniță et al. [10] 2021	3	Antenatal	NR	Unilateral multicystic dysplastic kidney	No	No	-	1.35 Mb deletion ^e^
			Antenatal	NR	Megabladder	No	No	Single umbilical artery, choroid plexus cyst, the possible absence of ductus arteriosus	1.31 Mb deletion ^e^
			7yr 5m	M	Bilateral hydronephrosis	No	No	Developmental delay, ASD, mild ID, language and speech disorders, poor fine and gross motor skills, social skills deficit, dysmorphic features, bilateral cryptorchidism, hypotonia, hirsutism, skeletal defects	1.80 Mb deletion ^f^
USA	Bustamante et al. [7] 2020	2	2yr 9m	M	Bilateral dysplastic kidneys	No	No	MODY5	1.43 Mb deletion
			1yr 10m	M	Bilateral dysplastic kidneys and left renal cysts	No	No	MODY5, global developmental delay.	1.5 Mb deletion ^g^
USA	Perosevic et al. [4] 2020	1	43	M	Ureteropelvic junction obstruction	No	Yes	Mild cognitive impairment, marfanoid habitus.	Deletion

The onset of diagnosis differs from one patient to another depending on the presentation. However, most of the cases were diagnosed in late adulthood. Late diagnosis might be due to the absence of the well-known features of the 17q12 syndrome or misdiagnosing MODY5 [5].

Though our patient doesn't have any dysmorphic features noticed at presentation or during her follow-up visits, dysmorphic features have been reported in the literature which includes right posterior plagiocephaly, facial asymmetry, narrow or high forehead, long and narrow appearing face, small chin, high and narrow palate, spare eyebrows, deep-set eyes, hypotelorism or hypertelorism, down slanting fissure, wide fleshy auricular pavilions, protruding cheekbones, anteverted nostrils, long philtrum, thin upper lips, a tuft of hair the neck to the left parotid region, abnormal teeth, and single plantar crease [1-3,10,15,16].

Our patient has shown a structural renal anomaly which is a solitary right kidney with left renal agenesis that has been diagnosed during antenatal ultrasound screening. Structural and functional renal anomalies represent the most frequent cardinal presentation of the affected individuals with 17q12 recurrent deletion syndrome [1,2,6,7]. The congenital anomalies of kidneys and urinary tract (CAKUT) are associated with HNF1B mutation and include multicystic kidneys, renal dysplasia, renal hypoplasia, renal agenesis, hydronephrosis, hydroureter, pyelectasis, duplicated collecting system, vesicoureteric reflux and horseshoe kidney [2]. Renal cystic dysplasia is the common major anomaly reported among 17q12 deletion syndrome patients [2,6]. The onset and severity of the presentation vary and can be detected during antenatal ultrasound screening, an amniotic fluid sampling or it manifests later in life [2,3,11,16,17]. Amniotic fluid sampling and analysis is an important step to be considered in pregnancies with renal anomalies to reach the diagnosis, as seen in two reported cases by Țuțulan-Cunițăa et al [10]. Similar to our patient, Chen et al. have reported a case of an asymptomatic carrier pregnant mother in which renal anomalies were detected during antenatal screening and the genetic testing confirmed 17q12 microdeletion encompassing HNF1B and LXH1 [1-3,11,14]. This highlights the importance of genetic counseling and screening for this syndrome in families with variable renal phenotype expression. Nakamura et al. have identified that genetic testing is the most accurate method to confirm doubts about any developmental renal disease, as it is important to distinguish between HNF1B mutation-related renal disease and autosomal recessive polycystic kidney disease (APRKD) [17].

Elevated liver enzymes were also noticed in our patient during her annual nephrology follow-up with a chief complaint of pruritis. However, gastrointestinal manifestations were less common features associated with 17q12 deletion syndrome [5] and the reported cases with elevated liver enzymes were asymptomatic [8,9,12,13].

The magnesium level changes are noticed to be within the normal values in our patient laboratory checkup tests done with every follow-up appointment. Magnesium impairment may occur in patients with 17q12 deletion syndrome and can be an atypical leading clue in the diagnosis of this syndrome. Chen et al. [5] and Perovsic et al. [4], determined that refractory hypomagnesemia is an important atypical predictor for 17q12 syndrome. Cheng et al. [5] reported a case of a 21-year-old female who was misdiagnosed initially with type 2 diabetes mellitus at age of 20 and refractory hypomagnesemia was the earliest clue for her diagnosis with MODY5 in the absence of well-known features of 17q12 syndrome [5], whereas Perosvic et al. [4] identified a case of a male patient who presented with ureteropelvic junction obstruction, chronic hydronephrosis, mild cognitive impairment, marfanoid features, and refractory hypomagnesemia.

The HNF1B gene is responsible for pancreatic development [2,5]. The pancreatic involvement findings include structural abnormalities, which most often are hypoplasia, atrophy, or agenesis of the body and tail [2]. Pancreatic atrophy and dysplasia have been shown in many patients with 17q12 deletion syndrome [5]. Exocrine pancreatic dysfunction is more common than pancreatic structural abnormalities [5]. During the ultrasound screening that has been done for our patient at the time of presentation, her pancreatic structure was normal.

In addition to pancreatic structural anomalies, 17q12 deletion syndrome is also a common cause of MODY5 [7,9,10], which is an autosomal dominant type of monogenic diabetes [5]. The presentation onset of MODY5 is usually at the age between 13 and 25 years of age [3,7], but it may occur in any age group [1]. Bustamante et al. have reported two cases where the patients presented early with MODY5 along with renal manifestations at the age of 22 and 33 months [7]. Although a family history of diabetes is not a helpful clue in the diagnosis of MODY5 [8,9], the diagnosis of MODY5 is usually delayed or misdiagnosed especially in absence of a family history of diabetes or well-known features of HNF1B disease [5,9], as seen in a Japanese case report by Omura et al. [9], where MODY5 was misdiagnosed earlier in life. Our patient's glucose levels and hemoglobin A1c are tested with every follow-up appointment and to date, the results show normal values. However, long-term follow-up is important as she might develop diabetes late on.

Furthermore, 17q12 deletion syndrome can affect the central nervous system and can be a cause of intellectual disability (ID), autistic features, cerebral anomaly, and developmental delay with or without behavioral problems [1,3]. Our patient had normal intellectual abilities but had language difficulties, especially in Arabic, when compared to students her age which indicates developmental delay. Most of the 17q12 deletion patients with HNF1B mutation are intellectually normal and not involved in neurodevelopment and neuropsychiatric features [1,3,5]. They have also identified that both forkhead box P2 (FOXP2) and acetyl-CoA carboxylase alpha (ACACA) genes were responsible prospectively for ID and behavioral disorders [1] whereas the LHX1 gene is involved in learning difficulties [3]. Palumbo et al. [1] described a case report of a patient with unusual HNF1B mutation-associated features; the patient had shown dysmorphic features and other mental impairments including ID, autistic features, serious speech delay, and obesity. George et al. [3] have identified that 17q12 deletion syndrome is associated with intellectual impairment, as he reported a case of a seven-year-old female with ADHD, learning difficulties, and normal renal features. A genetic study should be considered for any patients with learning difficulties and a positive family history of it [3].

Also, 17q12 deletion syndrome can affect the genital system which has been recently identified as a cause of Mayer-Rokitansky-Kuster-Hauser (MRKH) syndrome [1,3]. Bernardini et al. [15] reported two cases of MRKH syndrome, one of them associated with renal manifestation while the second case was of isolated MRKH syndrome; both were managed by laparoscopic corrective surgery.

The diagnosis of our patient was confirmed by genetic testing. Genetic studying is the gold standard test for the diagnosis of 17q12 deletion syndrome [1-17]. It is the most accurate study to diagnose patients with renal anomalies. [17]. Amniotic fluid sampling and analysis is an important step to be considered in pregnancies with renal anomalies to reach a diagnosis, as seen in two reported cases by Țuțulan-Cuniță et al [10]. There is no association found between the clinical features, gene contents, and the size of deletion yet.

This report scopes the importance of screening glucose, liver functions, and magnesium impairment in individuals with a solitary kidney, as this helps in an early diagnosis and management of this rare syndrome.

## Conclusions

While 17q12 deletion syndrome is a rare multisystemic affection condition that can be delayed in diagnosis according to the onset of presentation, early prenatal detection and accurate diagnosis are indispensable. Multidisciplinary team care and optimal parental counseling are important to plan the best management strategy and improve the child’s outcome. Further studies about this condition are required.
